# Cardiovascular Response of Aged Outpatients With Systemic Diseases During Tooth Extraction: A Single-Center Retrospective Observational Study

**DOI:** 10.3389/fpubh.2022.938609

**Published:** 2022-07-19

**Authors:** Jinjin Li, Zhiyan Tian, Shuqun Qi, Jiankang Zhang, Longjiang Li, Jian Pan

**Affiliations:** ^1^State Key Laboratory of Oral Diseases & National Clinical Research Center for Oral Diseases, Department of Oral and Maxillofacial Surgery, West China Hospital of Stomatology, Sichuan University, Chengdu, China; ^2^State Key Laboratory of Oral Diseases & National Clinical Research Center for Oral Diseases, Department of Head and Neck Oncology, West China Hospital of Stomatology, Sichuan University, Chengdu, China

**Keywords:** tooth extraction, aged patients, heart rate, blood pressure, multilevel model, systemic diseases

## Abstract

**Background:**

Aged people are maintaining many natural teeth due to improved oral health. However, compromised general health and poor oral hygiene habits at earlier ages resulted in poor status of preserved teeth. Therefore, tooth extraction is required in many aged people. More knowledge is needed because there are many risk factors during the surgery in frail aged adults. The aim of this study was to evaluate the cardiovascular response of such a population during tooth extraction and analyze risk factors to provide clinical guidance.

**Methods:**

A retrospective study was performed on aged patients with systemic diseases who underwent tooth extraction. Data regarding demographic profiles and cardiovascular parameters of heart rate and blood pressure were collected preoperative, when local anesthesia was administered, at the beginning of tooth extraction, 5 min after tooth extraction, and postoperative. The effects of risk factors, including age, sex, and systemic diseases on these parameters were analyzed with a multilevel model.

**Results:**

Heart rate (HR), systolic blood pressure (SBP), and diastolic blood pressure (DBP) of aged patients increased significantly when performing local anesthesia and tooth extraction. During the operation, the older patients (β = 2.011, *P* = 0.005) and the diabetics (β = 3.902, *P* < 0.0001) were associated with higher SBP, while those with more tooth extractions exhibited higher HR (β = 0.893, *P* = 0.007). Women patients showed both significantly elevated HR (β = 1.687, *P* < 0.0001) and SBP (β = 2.268, *P* < 0.0001). However, for coronary artery disease patients, HR (β = −2.747, *P* < 0.0001) and blood pressure [SBP (β = −4.094, *P* < 0.0001) and DBP (β = −0.87*, P* = 0.016)] were markedly lower than those of patients without a diagnosis of coronary artery disease.

**Conclusion:**

Cardiovascular response of aged outpatients with systemic diseases during tooth extraction is quite significant. Age, sex, systemic diseases, and the number of tooth extraction could be risk factors closely associated with cardiovascular response. The findings might provide safety guidance for dentists on tooth extraction in this population.

## Introduction

In China, people aged 60 years and older account for ~17.9% of the total population according to the latest demographic statistics (1). Among aged people, there is a high incidence of systemic diseases, such as hypertension, diabetes mellitus (DM), and coronary artery disease (CAD) (2–4). With the steadily increasing aged population, the number of aged people with systemic diseases is also increasing in dental visits. The oral health of people has improved in recent years, and many aged people still retain their natural teeth in old age (5, 6). However, in such a population, the decreased general health and dependence on care could easily lead to a poor oral condition (7–9). Therefore, tooth extraction in aged people with systemic diseases is becoming fairly common. Although tooth extraction is a frequent minor surgical procedure, the sudden increase in blood pressure (BP) and heart rate (HR) may still lead to severe complications and even death (10). Aged individuals with systemic diseases are generally fragile and susceptible to external stimuli, which inevitably increases their vulnerability to adverse events (11). Therefore, how to perform tooth extraction smoothly and safely in such patients is a great concern for dental surgeons.

Previous studies have demonstrated that the increase in BP during dental surgery cannot be predicted by the baseline BP (12, 13). The increase is not only related to the general condition of the target population but also to the mental stress and difficulty of tooth extraction and the use of local anesthetics (LA) during surgery (14–16). The monitoring of cardiovascular parameters, including HR and BP, during surgery procedures is an effective measure to assess risk and prevent adverse outcomes (17). This method is non-invasive and could provide a reliable valuable electrical activity index and guide the real-time processing, which is very useful for the prevention of severe cardiovascular complications, such as acute myocardial infarction and arrhythmia (18). Consequently, we assumed that the analysis of cardiovascular parameters in aged outpatients with systemic diseases during tooth extraction in a large sample could provide guidance for tooth extraction among such a population.

In this study, we retrospectively analyzed the changes in HR and BP during tooth extraction and the possible influencing factors involved in age, sex, and systemic diseases, aiming to determine risk factors and provide clinical support for dental extraction in this kind of patient.

## Materials and Methods

### Study Design

This retrospective observational study was conducted by collecting HR and BP data from aged patients with systemic diseases during tooth extraction. On the basis of age, sex, systemic diseases, and the number and difficulty of teeth extracted per time, risk factors influencing the cardiovascular response were assessed.

### Patient Selection and Data Collection

This study enrolled 3,044 aged outpatients (≥60 years) who received tooth extraction under electrocardiographic monitoring at West China Hospital of Stomatology at Sichuan University from January 2017 to December 2018. All patients signed the same questionnaire and received physician examinations by professional anesthesiologists. The questionnaire included demographics, age, sex, and systemic diseases. Systemic diseases included: (a) cardiovascular diseases: CAD, arrhythmia, pulmonary heart disease (PHD), myocardial infarction, and rheumatic heart disease; (b) hypertension: (SBP ≥140 mmHg and/or DBP ≥90 mmHg); (c) DM: [fasting food-glucose ≥ 7 mmol/L (more than 8 h) and/or 2-h postprandial blood sugar ≥ 11.1 mmol/L]; (d) cerebrovascular diseases: cerebral hemorrhage, cerebral infarction; (e) respiratory diseases: chronic obstructive pulmonary disease, pneumonectasis, and chronic bronchitis; (f) kidney diseases: nephritis and kidney neoplasms. Systemic diseases were diagnosed in line with guidelines for the prevention and treatment of corresponding diseases in China (2010 edition). All patients took their medications normally if needed. The criteria for inclusion included the following: (1) aged 60 years old and above; (2) experienced at least one tooth extraction; (3) complete medical records. Exclusion criteria included contraindications for tooth extraction and the absence of related medical data. Written informed consent was obtained from all study participants before dental extraction.

In view of the cardiovascular effects of epinephrine, the dental surgeons used two percent lidocaine hydrochloride without epinephrine for local anesthesia in all patients. The medical records mainly provided information on the number of tooth extraction, the reason for tooth extraction, the use of dental high-speed turbine handpieces, and the prognosis of the wound. The HR and BP were measured electronically by electrocardiograph monitoring and collected preoperatively, when local anesthesia was administered, at the beginning of tooth extraction, 5 min after the tooth extraction, and postoperative in all patients. An Access database and Excel system were used for data entry and sorting.

### Statistical Analysis

For repeated measurement variables (HR and BP), the data are shown as the mean ± standard deviation (X ± S), and multilevel linear model (MLM) data analysis was performed with SAS 9.4 software (19). We defined the patient as level 3, the number of tooth extraction as level 2, and different time points as level 1. The patient-level included the variables of age, sex, and systemic diseases. The number of tooth extraction ranged from one to five. The time points included preoperative, when local anesthesia was administered, the beginning of tooth extraction, 5 min after tooth extraction, and postoperative. Data hierarchies were assessed with a null model. To analyze the effect of multiple variables (age, sex, the number of tooth extraction per time, high-speed turbine handpieces, and systemic diseases) on the BP and HR during dental surgery, three-level random coefficient models were fitted in the study. The data were first analyzed with the univariate analysis, and variables with a value of *P* < 0.05 were further included in the multivariate analysis.

## Results

### Demographic Characteristics

**Figure 1:**
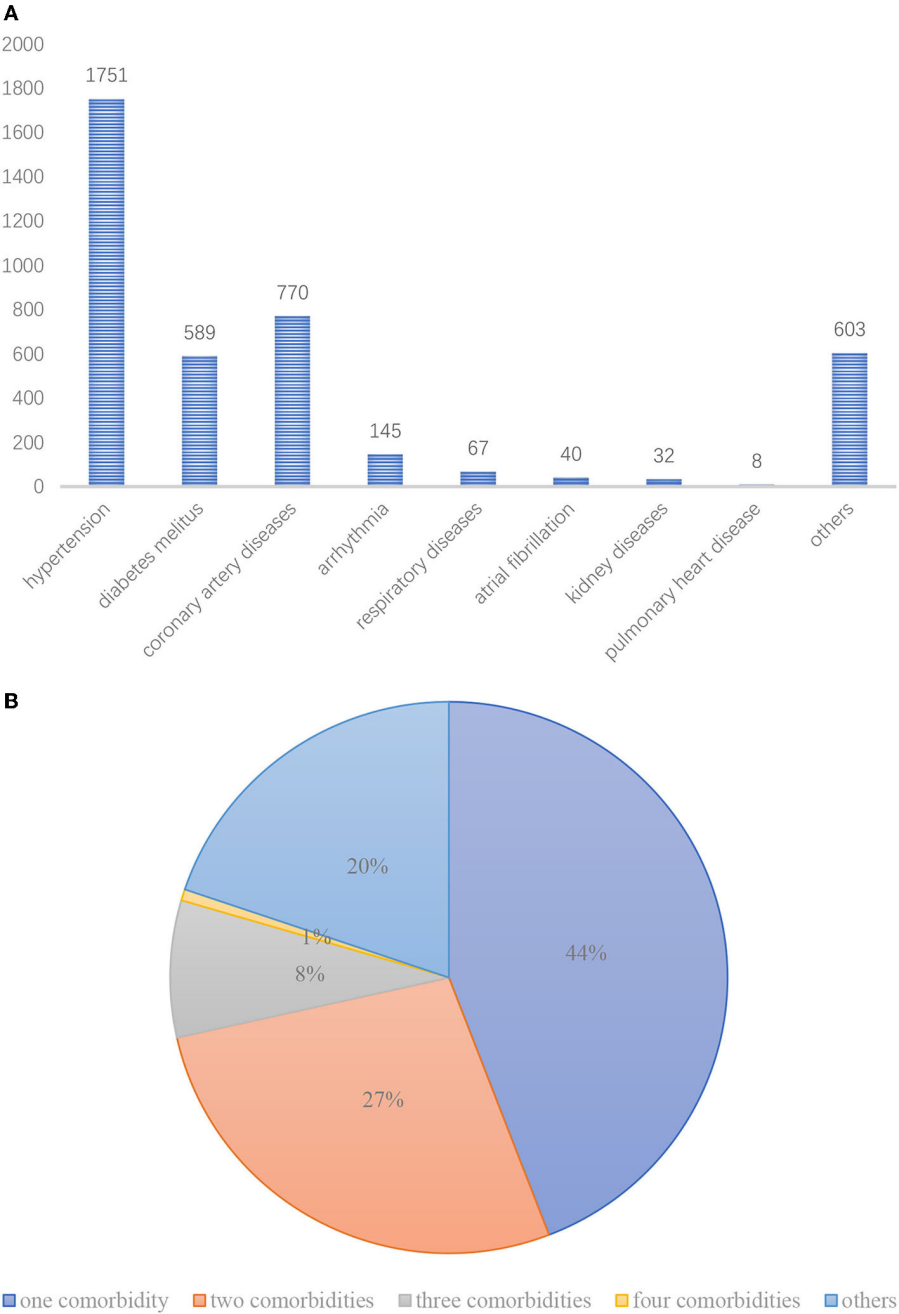
**(A)** Number of older patients with different systemic diseases. **(B)** The proportion of older patients with different systemic diseases.

A total of 3,044 patients with a median age of 74 (68, 80) years were included in this study. There were 1,347men (44.3%) and 1,697 women (55.7%). The number and proportion of patients with different systemic diseases are shown in Figures 1A,B. Among them, the most frequent systemic diseases were hypertension, CAD, DM, and arrhythmia, with proportions of 57.5, 25.3, 19.3, and 4.8%, respectively. According to age, the subjects were divided into three age groups: 60–69 years, 70–79 years, and 80–101 years. Our results showed that most patients were aged from 70 to 79 years old, accounting for 44.8% of the patients. It was followed by patients aged 60–69 years at 29.6%. These patients had a minimum of one tooth extracted and a maximum of 12 teeth extracted with the following distribution (1, 2.146, and 2, 12), where 2.146 represents the mean value and 2 represents the median value.

### Influence of Tooth Extraction on the Cardiovascular Response

In this study, the included patients underwent a total of 4,051 dental extractions. The reasons for tooth extraction were shown in the Supplementary Table 1. According to the monitoring records, the highest SBP was 243 mmHg, which was observed in a woman patient with hypertension and DM when administered local anesthesia. The highest DBP was 137 mmHg, which appeared in a woman patient with hypertension and CAD at the beginning of the operation. The highest HR, 166 beats/min, was also observed at the beginning of the operation in a woman patient with atrial fibrillation. Although some patients presented abnormal HR and BP values during the perioperative period, all the cases completed the surgery safely with the assistance of professional anesthesiologists at last. One week later, all the surgical wounds healed well.

The values of HR and BP in all patients at different time points during dental surgery are summarized in the Supplementary Tables 2–4. According to statistical analysis, the null model results of HR and BP showed that the data had a hierarchy structure (*P* < 0.05) and should be analyzed with a multilevel model (Supplementary Table 5). In the MLM results, the values of β represented coefficient estimates. When the value is more than zero, this means this kind of patient has higher HR or BP levels.

### Age, Sex and the Number of Tooth Extraction per Time

For HR, as shown in Table 1, the HR of older patients (70–80 years β = −1.972, *P* < 0.0001 and 80–101 years β = −3.083, *P* < 0.0001) was lower than that of patients in their sixties. In contrast, women patients (β = 1.687, *P* < 0.0001) and patients with 2 or more tooth extraction per time (β = 0.893, *P* = 0.007) had higher HR levels.

**Table 1:** Multilevel model of HR.

**Variable**	**HR**									
	**Univariate analysis**					**Multivariate analysis**				
	**β**	**SE**	***t***	**95% CI**	***P*-value**	**β**	**SE**	***t***	**95% CI**	***P*-value**
Intercept	–	–	–		–	67.213	0.701	95.85		<0.0001
**Age**										
(60, 70)	Ref	–	–		–	Ref	–	–		–
(70, 80)	−2.017	0.495	−4.08	−2.987, −1.047	<0.0001	−1.972	0.492	−4.01	−2.936, −1.00	<0.0001
(80, 101)	−3.315	0.563	−5.89	−4.418, −2.212	<0.0001	−3.083	0.563	−5.48	−4.186, −1.980	<0.0001
**Sex**										
Male	Ref	–	–		–	Ref	–	–		–
Female	2.321	0.421	5.52	1.496, 3.146	<0.0001	1.687	0.433	3.89	0.838, 2.536	<0.0001
**Systemic diseases**										
No	Ref	–	–		–	–	–	–		–
1	−0.272	0.495	−0.551	−1.242, 0.697	0.5820					
2 or more	−0.615	0.512	−1.200	−1.619, 0.389	0.2301	–	–	–		–
**Hypertension**										
No	Ref	–	–		–	–	–	–		–
Yes	0.132	0.422	0.31	−0.695, 0.959	0.754	–	–	–		–
**CAD**										
No	Ref	–	–		–	Ref	–	–		–
Yes	−2.365	0.474	−5	−3.294, −1.436	<0.0001	−2.747	0.486	−5.65	−3.700, −1.794	<0.0001
**DM**										
No	Ref	–	–		–	–	–	–		–
Yes	0.394	0.533	0.74	−0.651, 1.439	0.46	–	–	–		–
**Arrhythmia**										
No	Ref	–	–		–	Ref	–	–		–
Yes	−2.091	0.971	−2.15	−3.994, −0.188	0.03	−2.524	0.962	−2.62	−4.410, −0.638	0.009
**Dental high-speed turbine handpieces**										
No	Ref	–	–		–	–	–	–		–
Yes	−0.130	0.630	−0.21	−1.365, 1.105	0.84	-	–	–		–
**Number of tooth extraction per time**										
1	Ref	–	–		–	Ref	–	–		–
2 and more	0.803	0.330	2.43	0.156, 1.450	0.02	0.893	0.328	2.72	0.250, 1.536	0.007
**PHD**
No	Ref	–	–		–	–	–	–		–
Yes	6.659	4.063	1.64	−1.304, 14.622	0.10	–	–	–		–
**Atrial fibrillation**										
No	Ref	–	–		–	–	–	–		–
Yes	3.426	2.123	1.61	−0.735, 7.587	0.107	–	–	–		–
**Kidney diseases**										
No	Ref	–	–		–	–	–	–		–
Yes	0.116	2.067	0.06	−3.935, 4.167	0.96	–	–	–		–

*HR, heart rates; CAD, coronary artery disease; DM, Diabetes mellitus; PHD, pulmonary heart disease; β, coefficient estimates; SE, standard error; 95%CI, 95% confidence interval. Significant difference at P < 0.05, Ref, reference*.

For BP, the older patients showed significantly elevated SBP (β = 2.011, *P* = 0.005) and decreased DBP (β = −7.217, *P* < 0.0001), which was consistent with the variation trend of SBP (β = 2.268, *P* < 0.0001) and DBP (β = −1, *P* < 0.0001) in women, but there was no obvious difference in the number of tooth extraction at one time (Table 2).

**Table 2:** Multilevel model of SBP.

**Variable**	**SBP**									
	**Univariate analysis**					**Multivariate analysis**				
	**β**	**SE**	***t***	**95%CI**	***P*-value**	**β**	**SE**	***t***	**95%CI**	***P*-value**
Intercept	–	–	–		–	120.580	0.779	154.86		<0.0001
**Age**										
(60, 70)	Ref	–	–		–	Ref	–	–		–
(70, 80)	1.522	0.640	2.38	0.268, 2.776	0.018	1.139	0.616	1.85	−0.068, 2.346	0.065
(80, 101)	1.926	0.728	2.65	0.499, 3.353	0.008	2.011	0.707	2.84	0.625, 3.397	0.005
**Sex**										
Male	Ref	–	–		–	Ref	–	–		–
Female	2.073	0.542	3.82	1.011, 3.135	0.000	2.268	0.523	4.34	1.243, 3.293	<0.0001
**Systemic diseases**										
No	Ref	–	–		–	Ref	–	–		–
1	4.018	0.641	6.264	2.761, 5.275	<0.0001	−1.010	0.773	−1.306	−2.525, 0.505	0.1914
2 or more	6.564	0.664	9.885	5.263, 7.865	<0.0001	−2.243	1.137	−1.972	−4.472, −0.013	0.0487
**Hypertension**										
No	Ref	–	–		–	Ref	–	–		–
Yes	7.939	0.524	15.14	6.912, 8.966	<0.0001	9.941	0.820	12.13	8.334, 11.548	<0.0001
**CAD**										
No	Ref	–	–		–	Ref	–	–		–
Yes	−1.491	0.615	−2.42	−2.696, −0.286	0.015	−4.094	0.897	−4.56	−5.852, −2.336	<0.0001
**DM**										
No	Ref	–	–		–	Ref	–	–		–
Yes	4.118	0.682	6.04	2.781, 5.455	<0.0001	3.902	0.710	5.5	2.510, 5.294	<0.0001
**Dental high-speed turbine handpieces**										
No	Ref	–	–		–	–	–	–		–
Yes	−0.859	0.886	−0.97	−2.596, 0.878	0.332	–	–	–		–
**Number of tooth extraction per time**										
1	Ref	–	–		–	–	–	–		–
2 and more	0.648	0.460	1.41	−0.254, 1.550	0.158	–	–	–		–
**Kidney diseases**										
No	Ref	–	–		–	–	–	–		–
Yes	2.298	2.661	0.86	−2.918, 7.514	0.388	–	–	–		–

*SBP, systolic blood pressure; CAD, coronary artery disease; DM, Diabetes mellitus; β, coefficient estimates; SE, standard error; 95%CI, 95% confidence interval. Significant difference at P < 0.05, Ref, reference*.

### Systemic Diseases

For HR, the multilevel model revealed that CAD (β = −2.747, *P* < 0.0001) and arrhythmia (β = −2.524, *P* = 0.009) could cause a decreased HR compared to the patients without a diagnosis of CAD and arrhythmia during the dental surgery (Table 1).

For BP, hypertension patients showed higher SBP (β = 9.941, *P* < 0.0001) and DBP (β = 0.94, *P* = 0.003) than nonhypertensive patients. In contrast, patients with CAD had lower SBP (β = −4.094, *P* < 0.0001) and DBP (β = −0.87, *P* = *0.0*16). In DM, this population had higher SBP (β = 3.902, *P* < 0.0001) and lower DBP (β = −1.634, *P* < 0.0001;3 Tables 2, 3).

**Table 3:** Multilevel model of DBP.

**Variable**	**DBP**									
	**Univariate analysis**					**Multivariate analysis**				
	**β**	**SE**	***t***	**95%CI**	***P*-value**	**β**	**SE**	***t***	**95%CI**	***P*-value**
Intercept	–	–	–		–	73.511	0.813	90.41		<0.0001
**Age**										
(60, 70)	Ref	–	–		–	Ref	–	–		–
(70, 80)	−3.940	0.376	−10.48	−4.677, −3.203	<0.0001	−4.064	0.373	−10.9	−4.795, −3.333	<0.0001
(80, 101)	−6.899	0.428	−16.14	−7.738, −6.060	<0.0001	−7.217	0.427	−16.92	−8.054, −6.380	<0.0001
**Sex**										
Male	Ref	–	–		–	Ref	–	–		–
Female	−1.346	0.332	−4.06	−1.997, −0.695	<0.0001	−1.000	0.318	−6.24	0.377, 1.623	<0.0001
**Systemic diseases**										
No	Ref	–	–		–	Ref	–	–		–
1	1.199	0.394	3.041	0.426, 1.972	0.0024	0.008	0.469	0.017	−0.911, 0.927	0.9862
2 or more	0.235	0.408	0.576	−0.565, 1.035	0.5645	−0.142	0.690	−0.206	−1.494, 1.210	0.8369
**Hypertension**										
No	Ref	–	–		–	Ref	–	–		–
Yes	0.691	0.333	2.08	0.038, 1.344	0.038	0.940	0.321	2.93	0.311, 1.569	0.003
**CAD**										
No	Ref	–	–		–	Ref	–	–		–
Yes	−0.737	0.376	−1.96	−1.474, 0.000	0.050	−0.870	0.360	−2.42	−1.576, −0.164	0.016
**DM**										
No	Ref	–	–		–	Ref	–	–		–
Yes	−1.586	0.419	−3.79	−2.407, −0.765	0.000	−1.634	0.402	−4.06	−2.422, −0.846	<0.0001
**Dental high-speed turbine handpieces**										
No	Ref	–	–		–	–	–	–		–
Yes	0.316	0.529	0.6	−0.721, 1.353	0.550	–	–	–		–
**Number of tooth extraction per time**										
1	Ref	–	–		–	–	–	–		–
2 and more	0.178	0.275	0.64	−0.361, 0.717	0.519	–	–	–		–
**Kidney diseases**										
No	Ref	–	–		–	–	–	–		–
Yes	−0.583	1.628	−0.36	−3.774, 2.608	0.721	–	–	–		–

*DBP, diastolic blood pressures; CAD, coronary artery disease; DM, Diabetes mellitus; β, coefficient estimates; SE, standard error; 95%CI, 95% confidence interval. Significant difference at P < 0.05, Ref, reference*.

## Discussion

In this retrospective study, we investigated the changes in HR and BP during tooth extraction in aged people with systemic diseases and analyzed some influencing factors. According to the univariate results, we found that various systemic diseases and other factors, including age, sex, and the number and difficulty of tooth extraction per time exert different effects on HR and BP during dental extraction in aged, medically compromised patients. After performing the multivariate analysis, we observed that women, diabetic patients, and older patients showed significantly increased SBP. Patients with more tooth extraction and women had a higher HR. In contrast, patients with CAD exhibited lower HR and BP.

Control of HR and BP plays a vital role in preventing cardiovascular complications during dental surgery in aged patients with systemic diseases (20–23). In dental extraction, many factors can affect HR and BP, particularly pain. LA is a factor closely associated with pain control. Regarding the LA, whether epinephrine, a vasoconstrictor, should be used is a question that has been repeatedly discussed (24–26). The adverse cardiovascular effects associated with the epinephrine are unequivocal, especially in aged and or medically comprised adults. Although many recent studies have reported that epinephrine does not cause an increase in HR and BP, the clinical application still needs to be combined with the actual situation of the outpatients (10, 27). In our study, most of the teeth that needed to be extracted were simple teeth, and the surgery duration was short. Importantly, the included patients were aged adults with various systemic diseases. Therefore, LA was administered with two percent lidocaine hydrochloride without epinephrine. Given the results regarding changes in HR and BP without its use, it is no wonder that this is an issue worthy of further study.

According to the reasons for tooth extraction, we found periodontal disease, especially periodontitis was a common cause of tooth extraction. It is well-known inflammation can also alter the action of LA interfering with adequate anesthesia (28). Although the extracted teeth in our study had no or mild inflammation, the effect on LA should still be considered. Petersilka et al. reported that applying local anesthesia may offer a safe and effective way to achieve pain-free therapy in periodontal patients (29). Additionally, according to the clinical observation, the mild inflammation had no significant effect on local anesthesia. However, this issue also needs further investigation.

Dental anxiety and fear are common reactions to dental treatment (30, 31). They can be described as an emotional state of fear of dental stimuli. There are some studies that have investigated the relationship between dental anxiety and hemodynamic changes in dental patients, which indicated that psychological and physical stress due to anxiety and pain stimuli have some cardiovascular effects (14, 17, 32). Regarding the influencing factors, injection pain and tooth turbine vibration were considered the major source of anxiety. Furthermore, sex has also been reported to be associated with dental anxiety. Women are reported to be more anxious in response to dental treatment than men (33, 34). In addition, women had a lower pain threshold and tolerance. These findings supported sex differences in the variations in HR and SBP in our study. Interestingly, the rate-pressure product (RPP) of HR and SBP is used to estimate myocardial oxygen consumption. In our study, both the increase in HR and SBP in women patients resulted in an elevation of RPP, which probably means that women are more susceptible to developing myocardial ischemia than men during tooth extraction. Therefore, these results suggested that the attenuation of anxiety and stress is essential and beneficial for dental patients, especially women.

The risk relationship between systemic diseases and tooth extraction has been explored in many dental practices. However, the cardiovascular impact of each disease is not determined clearly. In our study, the increase in BP was greater in aged patients with hypertension, which is consistent with previous reports (16, 35). However, patients with CAD showed a significantly lower HR during dental extraction than patients without a diagnosis of CAD. HR is often considered a key risk factor for adverse outcomes in patients with CAD. Its reduction is a recognized strategy to prevent myocardial ischemia (36, 37). According to previous studies, the functional mechanism of beta-blockers is decreasing HR (38, 39). Therefore, medication may be one of the reasons for the HR reduction during tooth extraction in patients with CAD. The result needs to be validated prospectively. DM is a disease closely related to dentistry. Although many previous studies have discussed the relationship between tooth loss and blood glucose levels, the variation in BP during tooth extraction in aged patients with DM has not yet been reported. According to our analysis, the elevation of SBP is significantly higher in aged patients with DM than in those without DM. It was reported that hypertension and DM were closely related due to similar pathophysiological factors including obesity, dyslipidemia, and atherosclerosis (40). Thus, DM could be exacerbated by hypertension to cause cardiovascular mortality and morbidity. The obvious increase in SBP during dental surgery in aged DM patients is likely due to chronic cardiovascular damage caused by DM. Of course, the impact of systemic disease is very complex, and more studies with large sample sizes are needed to explore this issue.

There are some strengths in our study. First, we included a relatively large sample of aged people undergoing tooth extraction. Second, five-time points for recording HR and BP are also relatively reasonable and can basically reflect intraoperative changes. Third, the use of multilevel model analysis methods further enhances the reliability and accuracy of the results. Furthermore, various risk factors were explored in our study. Therefore, these results can provide some guidance for clinical tooth extraction in an older population. However, there are also limitations in our study. First, the medications, we did not obtain enough data to assess the effects of these medications. Second, the ECG analysis was very valuable, but this section was not fully recorded in our available data.

## Conclusion

Based on our results, the variations in HR and BP during the tooth extraction showed significant differences among different aged patients. The influencing risk factors mainly included physical condition (systemic diseases: hypertension, DM, and CAD) and clinical characteristics (age, sex, the number of tooth extraction per time). Our findings might aid clinicians in predicting the possible variation in HR and BP and assessing the risk of tooth extraction in the aged patients before the surgery. However, further studies are needed to verify our findings.

## Data Availability Statement

The original contributions presented in the study are included in the article/ Supplementary Material, further inquiries can be directed to the corresponding authors.

## Ethics Statement

This retrospective study was approved by approved by the Ethic Committee of West China hospital of Stomatology in Sichuan University (WCHSIRB-D-2017-115). The patients/participants provided their written informed consent to participate in this study.

## Author Contributions

JL and ZT collected and interpreted data, and drafted the manuscript. SQ and JZ critically reviewed the manuscript. LL critically revised the manuscript. JP designed the experiment and critically reviewed the manuscript. All authors agree to be accountable for the study.

## Funding

This study was funded by Research and Develop Program, West China Hospital of Stomatology Sichuan University, NO. LCYJ2019-1.

## Conflict of Interest

The authors declare that the research was conducted in the absence of any commercial or financial relationships that could be construed as a potential conflict of interest.

## Publisher's Note

All claims expressed in this article are solely those of the authors and do not necessarily represent those of their affiliated organizations, or those of the publisher, the editors and the reviewers. Any product that may be evaluated in this article, or claim that may be made by its manufacturer, is not guaranteed or endorsed by the publisher.

## References

[1] National Bureau of Statistics of China. China statistical yearbook. (2019). Available online at: http://www.stats.gov.cn/tjsj/ndsj/2019/indexeh.htm (accessed April 20, 2020).

[2] Liu S Wang W Zhang J He Y Yao C Zeng Z. Prevalence of diabetes and impaired fasting glucose in Chinese adults, China National Nutrition and Health Survey, 2002. Prev Chronic Dis. (2011) 8:A13. 10.3109/10903127.2010.525298 21159225 PMC3044024

[3] Wu F Guo Y Kowal P Jiang Y Yu M Li X. Prevalence of major chronic conditions among older Chinese adults: the Study on Global AGEing and adult health (SAGE) wave 1. PLoS ONE. (2013) 8:e74176. 10.1371/journal.pone.0074176 24069278 PMC3775793

[4] Mendis S Davis S Norrving B. Organizational update: The world health organization global status report on noncommunicable diseases 2014; one more landmark step in the combat against stroke and vascular disease. Stroke. (2015) 46:e121–2. 10.1161/STROKEAHA.113.003377 25873596

[5] Chu BF Zhang Y Liu HC. [Minimal intervention dentistry: a vision of caries management for older patients. II] *Hua Xi Kou Qiang Yi Xue Za Zhi*. (2010) 28:9–12. 10.3969/j.issn.1000-1182.2010.01.003 20337065

[6] He S Thomson WM. An oral epidemiological comparison of Chinese and New Zealand adults in 2 key age groups. Community Dent Oral Epidemiol. (2018) 46:154–60. 10.1111/cdoe.12348 29094770

[7] MacEntee MI Donnelly LR. Oral health and the frailty syndrome. Periodontol 2000. (2016) 72:135–41. 10.1111/prd.12134 27501496

[8] Gibney JM Naganathan V Lim M. Oral health is essential to the well-being of older people. Am J Geriatr Psychiatry. (2021) 29:1053–7. 10.1016/j.jagp.2021.06.002 34246517

[9] Koistinen S Stahlnacke K Olai L Ehrenberg A Carlsson E. Older people's experiences of oral health and assisted daily oral care in short-term facilities. BMC Geriatr. (2021) 21:388. 10.1186/s12877-021-02281-z 34176481 PMC8237451

[10] Abu-Mostafa N Aldawssary A Assari A Alnujaidy S Almutlaq A. A prospective randomized clinical trial compared the effect of various types of local anesthetics cartridges on hypertensive patients during dental extraction. J Clin Exp Dent. (2015) 7:e84–8. 10.4317/jced.51534 25810849 PMC4368025

[11] Abellan VKG Rolland Y Houles M Gillette-Guyonnet S Soto M Vellas B. The assessment of frailty in older adults. Clin Geriatr Med. (2010) 26:275–86. 10.1016/j.cger.2010.02.002 20497846

[12] Matsumura K Miura K Takata Y Kurokawa H Kajiyama M Abe I. Changes in blood pressure and heart rate variability during dental surgery. Am J Hypertens. (1998) 11:1376–80. 10.1016/S0895-7061(98)00157-5 9832183

[13] Mani S Chen Y Elasy T Clayton W Denny J. Type 2 diabetes risk forecasting from EMR data using machine learning. AMIA Annu Symp Proc. (2012) 2012:606–15. 23304333 PMC3540444

[14] Liau FL Kok SH Lee JJ Kuo RC Hwang CR Yang PJ. Cardiovascular influence of dental anxiety during local anesthesia for tooth extraction. Oral Surg Oral Med Oral Pathol Oral Radiol Endod. (2008) 105:16–26. 10.1016/j.tripleo.2007.03.015 17656135

[15] Neves RS Neves IL Giorgi DM Grupi CJ Cesar LA Hueb W. Effects of epinephrine in local dental anesthesia in patients with coronary artery disease. Arq Bras Cardiol. (2007) 88:545–51. 10.1590/S0066-782X2007000500008 17589629

[16] Tsuchihashi T Takata Y Kurokawa H Miura K Maruoka Y Kajiyama M. Blood pressure response during dental surgery. Hypertens Res. (1996) 19:189–94. 10.1291/hypres.19.189 8891747

[17] Lu P Gong Y Chen Y Cai W Sheng J. Safety analysis of tooth extraction in elderly patients with cardiovascular diseases. Med Sci Monit. (2014) 20:782–8. 10.12659/MSM.890131 24819043 PMC4031223

[18] Serhani MA T EKH Ismail H Nujum NA. ECG monitoring systems: Review, architecture, processes, and key challenges. Sensors. (2020) 20:1796. 10.3390/s20061796 32213969 PMC7147367

[19] Behm JE Edmonds DA Harmon JP Ives AR. Multilevel statistical models and the analysis of experimental data. Ecology. (2013) 94:1479–86. 10.1890/12-2005.1 23951708

[20] Kubota K Yamaga E Ueda K Inokoshi M Minakuchi S. Comparison of cardiovascular response between patients on warfarin and hypertensive patients not on warfarin during dental extraction. Clin Oral Investig. (2021) 25:2141–50. 10.1007/s00784-020-03526-8 32808177

[21] Safar ME. Systolic blood pressure, pulse pressure and arterial stiffness as cardiovascular risk factors. Curr Opin Nephrol Hypertens. (2001) 10:257–61. 10.1097/00041552-200103000-00015 11224702

[22] Gosmanova EO Mikkelsen MK Molnar MZ Lu JL Yessayan LT Kalantar-Zadeh K. Association of systolic blood pressure variability with mortality, coronary heart disease, stroke, and renal disease. J Am Coll Cardiol. (2016) 68:1375–86. 10.1016/j.jacc.2016.06.054 27659458 PMC5117818

[23] Ogunlewe MO James O Ajuluchukwu JN Ladeinde AL Adeyemo WL Gbotolorun OM. Evaluation of haemodynamic changes in hypertensive patients during tooth extraction under local anaesthesia. West Indian Med J. (2011) 60:91–5. 10.1590/S0100-736X2012001200024 21809720

[24] Jage J. Circulatory effects of vasoconstrictors combined with local anesthetics. Anesth Pain Control Dent. (1993) 2:81–6. 8219930

[25] Becker DE Reed KL. Local anesthetics: Review of pharmacological considerations. Anesth Prog. (2012) 59:90–101, 102–3. 10.2344/0003-3006-59.2.90 22822998 PMC3403589

[26] Laragnoit AB Neves RS Neves IL Vieira JE. Locoregional anesthesia for dental treatment in cardiac patients: a comparative study of 2% plain lidocaine and 2% lidocaine with epinephrine (1:100,000). Clinics. (2009) 64:177–82. 10.1590/S1807-59322009000300005 19330241 PMC2666461

[27] Guimaraes CC Lopes LC Bergamaschi CC Ramacciato JC Silva MT Araujo JO. Local anaesthetics combined with vasoconstrictors in patients with cardiovascular disease undergoing dental procedures: Systematic review and meta-analysis. BMJ Open. (2021) 11:e44357. 10.1136/bmjopen-2020-044357 34266837 PMC8286772

[28] Ueno T Tsuchiya H Mizogami M Takakura K. Local anesthetic failure associated with inflammation: verification of the acidosis mechanism and the hypothetic participation of inflammatory peroxynitrite. J Inflamm Res. (2008) 1:41–8. 10.2147/JIR.S3982 22096346 PMC3218719

[29] Petersilka GJ Arweiler NB Otto J Wittig T. Non-interventional study to collect data for the application of lidocaine gel 2% during scaling and root planing and professional mechanical plaque removal. Clin Oral Investig. (2019) 23:551–8. 10.1007/s00784-018-2468-0 29717361 PMC7735999

[30] Brand HS Gortzak RA Abraham-Inpijn L. Anxiety and heart rate correlation prior to dental checkup. Int Dent J. (1995) 45:347–51. 8666460

[31] Ng SK Stouthard ME Keung LW. Validation of a Chinese version of the dental anxiety inventory. Community Dent Oral Epidemiol. (2005) 33:107–14. 10.1111/j.1600-0528.2004.00199.x 15725173

[32] Gadve VR Shenoi R Vats V Shrivastava A. Evaluation of anxiety, pain, and hemodynamic changes during surgical removal of lower third molar under local anesthesia. Ann Maxillofac Surg. (2018) 8:247–53. 10.4103/ams.ams_216_18 30693240 PMC6327800

[33] Emery CF Stoney CM Thayer JF Williams D Bodine A. Sex and family history of cardiovascular disease influence heart rate variability during stress among healthy adults. J Psychosom Res. (2018) 110:54–60. 10.1016/j.jpsychores.2018.04.011 29764606 PMC5966826

[34] Caltabiano ML Croker F Page L Sklavos A Spiteri J Hanrahan L. Dental anxiety in patients attending a student dental clinic. BMC Oral Health. (2018) 18:48. 10.1186/s12903-018-0507-5 29558935 PMC5859659

[35] Agani ZB Benedetti A Krasniqi VH Ahmedi J Sejfija Z Loxha MP. Cortisol level and hemodynamic changes during tooth extraction at hypertensive and normotensive patients. Med Arch. (2015) 69:117–22. 10.5455/medarh.2015.117-122 26005263 PMC4429991

[36] Ferrari R Fox K. Heart rate reduction in coronary artery disease and heart failure. Nat Rev Cardiol. (2016) 13:493–501. 10.1038/nrcardio.2016.84 27226153

[37] Tanna MS Messerli FH Bangalore S. Stable coronary artery disease: are there therapeutic benefits of heart rate lowering? J Hypertens. (2019) 37:1112–8. 10.1097/HJH.0000000000002041 30676481

[38] Fox K Borer JS Camm AJ Danchin N Ferrari R Lopez SJ. Resting heart rate in cardiovascular disease. J Am Coll Cardiol. (2007) 50:823–30. 10.1016/j.jacc.2007.04.079 17719466

[39] Kotecha D Flather MD Altman DG Holmes J Rosano G Wikstrand J. Heart rate and rhythm and the benefit of Beta-Blockers in patients with heart failure. J Am Coll Cardiol. (2017) 69:2885–96. 10.1016/j.jacc.2017.04.001 28467883

[40] Petrie JR Guzik TJ Touyz RM. Diabetes, hypertension, and cardiovascular disease: clinical insights and vascular mechanisms. Can J Cardiol. (2018) 34:575–84. 10.1016/j.cjca.2017.12.005 29459239 PMC5953551

