# Supplementary material for: Cardiovascular Response of Aged Outpatients With Systemic Diseases During Tooth Extraction: A Single-Center Retrospective Observational Study
Source: Front Public Health. 2022 Jul 19;10:938609. doi: 10.3389/fpubh.2022.938609 (PMC9344048; doi:10.3389/fpubh.2022.938609)
Supplement: Supplementary file 1 [file Table_1.pdf]

Supplementary Table 1 The reasons and proportion of tooth extraction

Extraction reasons	The proportion
Caries	72.1%
Periodontal disease	23.2%
Pre-prosthetic	3.7%
Dental trauma	0.2%
Patient request	0.8%

Supplementary Table 2 HR of aged patients at different time points ($\bar{X} \pm S$)

Variable	Number	Preoperative	Anesthesia	Tooth extraction Beginning	5mins after tooth extraction	Postoperative
Age						
[60,70)	1160	75.86±12.25	80.7±13.01	81.8±13.19	81.25±12.88	76.1±10.91
[70,80)	1837	74.08±11.85	79.07±12.86	80.04±13.00	79.28±12.62	74.53±10.57
[80,101]	1054	72.97±11.2	77.59±12.16	78.17±12.41	77.77±12.11	73.63±10.52
Sex						
Male	1818	73.34±12.03	77.94±12.85	78.67±12.91	78.03±12.49	73.54±10.71
Female	2233	75.09±11.64	80.15±12.63	81.18±12.92	80.6±12.63	75.73±10.59
Dental high-speed turbine handpieces						
No	3780	74.37±11.92	79.24±12.86	80.11±13.05	79.46±12.68	74.78±10.75
Yes	271	73.37±10.78	78.01±11.49	79.35±11.89	79.29±12.03	74.32±9.85
Number of tooth extraction per time						
1	1868	74.33±11.69	79.01±12.58	79.81±12.75	79.28±12.52	74.58±10.60
2 or more	2183	74.28±11.99	79.28±12.94	80.27±13.16	79.60±12.73	74.89±10.78
Hypertension						
No	1758	74.36±11.67	79.11±12.43	80.02±12.71	79.32±12.38	74.58±10.45
Yes	2293	74.26±11.99	79.19±13.03	80.09±13.17	79.54±12.82	74.87±10.88
CAD						
No	3036	74.93±11.84	79.83±12.69	80.67±12.92	80.03±12.5	75.27±10.54
Yes	1015	72.41±11.66	77.14±12.81	78.23±12.97	77.72±12.87	73.16±11.01

Arrhythmia						
No	3866	74.37±11.71	79.23±12.54	80.14±12.77	79.53±12.43	74.8±10.52
Yes	185	72.96±14.41	77.57±16.94	78.21±16.67	77.82±16.16	73.69±13.81
PHD						
No	4039	74.28±11.84	79.13±12.76	80.03±12.95	79.43±12.61	74.73±10.68
Yes	12	80.00±12.36	86.08±14.99	88.08±17.6	86.5±17.35	80.25±13.34
Kidney diseases						
No	4009	74.32±11.86	79.17±12.78	80.06±12.99	79.45±12.65	74.75±10.71
Yes	42	72.88±11.15	77.88±11.81	79.90±11.52	79.26±11.34	74.10±9.81
DM						
No	3289	74.1±11.91	79.02±12.88	79.99±13.16	79.38±12.79	74.64±10.81
Yes	762	75.16±11.56	79.76±12.31	80.36±12.15	79.74±11.90	75.21±10.19
Atrial fibrillation						
No	4011	74.28±11.83	79.11±12.7	79.98±12.82	79.38±12.51	74.71±10.65
Yes	40	76.18±13.31	83.9±17.98	87.98±22.89	86.33±20.81	78.7±14.38
Total	4051	74.3±11.85	79.16±12.77	80.06±12.97	79.45±12.63	74.74±10.70

Abbreviations: HR, heart rate; CAD, coronary artery disease; PHD, pulmonary heart disease; DM, Diabetes mellitus.

Supplementary Table 3 SBP of aged patients at different time points ($\bar{X} \pm S$)

Variable	Number	Preoperative	Anesthesia	Tooth extraction Beginning	5mins after tooth extraction	Postoperative
Age						
[60,70)	1160	139.38±16.69	148.64±18.75	149.95±18.21	149.02±17.17	140.13±14.35
[70,80)	1837	141.1±16.36	150.48±17.57	151.65±17.24	150.37±16.17	141.66±13.71
[80,101]	1054	141.74±16.23	150.79±17.65	151.78±17.83	150.82±17.12	142.21±14.15
Sex						
Male	1818	139.8±16.58	148.44±17.43	149.92±17.72	148.96±16.76	140.5±14.27
Female	2233	141.57±16.29	151.33±18.27	152.24±17.6	151.03±16.64	142.07±13.79
Dental high-speed turbine handpieces						
No	3780	140.89±16.47	150.17±18	151.19±17.74	150.05±16.68	141.39±14.03
Yes	271	139.15±16.06	148.14±17.3	151.25±17.06	150.85±17.31	141.01±14.11
Number of tooth extraction per time						
1	1868	141.46±16.43	150.18±17.83	151.34±17.53	150.1±16.45	141.2±13.97
2 or more	2183	140.19±16.43	149.91±18.07	151.08±17.83	150.11±16.96	141.51±14.08

Hypertension						
No	1758	135.98±16.32	144.92±17.63	146.42±17.37	145.5±16.51	137.32±14.11
Yes	2293	144.45±15.57	153.95±17.21	154.86±17.05	153.63±16.02	144.47±13.15
CAD						
No	3036	141.11±16.48	150.38±18.02	151.41±17.78	150.35±16.86	141.53±14.1
Yes	1015	139.78±16.3	148.99±17.74	150.55±17.42	149.35±16.3	140.87±13.8
Arrhythmia						
No	3866	140.86±16.48	150.15±17.99	151.4±17.74	150.25±16.69	141.47±14.03
Yes	185	138.88±15.58	147.48±17.18	146.92±15.95	147.07±17.21	139.11±13.81
PHD						
No	4039	140.79±16.43	150.05±17.95	151.22±17.69	150.13±16.71	141.38±14.02
Yes	12	134.67±20.12	143.33±19.03	143.83±17.93	141.67±19.07	136.08±16.74
Kidney diseases						
No	4009	140.74±16.44	150±17.96	151.15±17.69	150.07±16.71	141.33±14.04
Yes	42	143.88±16.46	152.93±17.57	156.05±17.17	153.17±17.72	144.31±12.78
DM						
No	3289	139.93±16.43	149.03±17.74	150.36±17.67	149.32±16.65	140.74±14.09
Yes	762	144.42±16.02	154.37±18.24	154.8±17.34	153.46±16.62	144.06±13.47
Total	4051	140.77±16.44	150.03±17.96	151.2±17.69	150.1±16.72	141.37±14.03

Abbreviations: SBP, systolic blood pressure; CAD, coronary artery disease; PHD, pulmonary heart disease; DM, Diabetes mellitus.

Supplementary Table 4 DBP of aged patients at different time points ($\bar{X} \pm S$)

Variable	Number	Preoperative	Anesthesia	Tooth extraction Beginning	5mins after tooth extraction	Postoperative
Age						
[60,70)	1160	76.87±10.65	79.08±11.09	78.9±10.61	78.21±9.75	74.47±8.15
[70,80)	1837	72.4±10.56	74.61±11.54	74.75±11.21	74.24±10.16	71.23±8.21
[80,101]	1054	69.41±10.63	71.6±11.66	71.35±11.01	71.54±10.25	68.75±8.66
Sex						
Male	1818	73.67±11.04	75.61±11.67	75.92±11.33	75.53±10.44	72.2±8.79
Female	2233	72.28±10.86	74.7±11.85	74.35±11.3	73.98±10.26	70.95±8.36
Dental high-speed turbine handpieces						
No	3780	72.9±11.03	75.17±11.84	75.03±11.36	74.65±10.4	71.46±8.6
Yes	271	72.89±9.98	74.31±10.84	75.34±11.04	75.07±10.01	72.17±8.33

Number of tooth extraction per time						
1	1868	73.01±11.1	75.18±11.99	75.32±11.40	74.73±10.37	71.6±8.78
2 and more	2183	72.81±10.85	75.05±11.6	74.83±11.28	74.63±10.37	71.44±8.41
Hypertension						
No	1758	72.52±10.25	74.54±11.18	74.6±10.69	74.23±9.5	71.22±8.01
Yes	2293	73.2±11.48	75.54±12.2	75.4±11.8	75.02±10.98	71.74±8.99
CAD						
No	3036	73.07±11.03	75.37±11.87	75.24±11.43	74.81±10.38	71.68±8.61
Yes	1015	72.39±10.74	74.33±11.48	74.5±11.05	74.28±10.34	71.02±8.49
Arrhythmia						
No	3866	72.91±10.93	75.1±11.75	75.11±11.31	74.69±10.33	71.51±8.57
Yes	185	72.67±11.68	75.22±12.44	73.97±11.88	74.43±11.22	71.48±8.89
PHD						
No	4039	72.89±10.96	75.09±11.77	75.04±11.33	74.67±10.36	71.51±8.57
Yes	12	76.83±13.52	79.75±15.06	79.33±14.09	78±13.41	71.58±12.71
Kidney diseases						
No	4009	72.91±10.94	75.13±11.77	75.05±11.31	74.68±10.35	71.52±8.58
Yes	42	72.57±12.98	72.57±12.41	75.29±13.75	74.07±12.1	70.36±8.58
DM						
No	3289	73.12±10.99	75.38±11.83	75.3±11.36	74.98±10.41	71.76±8.59
Yes	762	71.97±10.8	73.96±11.47	73.98±11.19	73.37±10.1	70.44±8.44
Total	4051	72.9±10.96	75.11±11.78	75.05±11.34	74.68±10.37	71.51±8.58

Abbreviations: DBP, diastolic blood pressure; CAD, coronary artery disease; PHD, pulmonary heart disease; DM, Diabetes mellitus.

Supplementary Table 5 The null model of HR, SBP and DBP

Effects	HR				SBP				DBP			
	β	SE	Z	P	β	SE	Z	P	β	SE	Z	P
Fixed effect	77.665	0.210	369.31	<0.0001	146.98	0.2704	543.47	<0.0001	73.961	0.166	446.88	<.0001
Random effect												
Level 1 (subject)	102.370	3.732	27.43	<0.0001	145.07	6.4969	22.33	<0.0001	57.412	2.377	24.15	<.0001
Level 2 (times)	34.207	1.699	20.13	<0.0001	78.4353	4.0377	19.43	<0.0001	24.234	1.352	17.93	<.0001
Level 3 (each time point)	19.992	0.222	90.01	<0.0001	72.4876	0.8053	90.01	<0.0001	33.776	0.375	90.01	<.0001
-2LL	131760.3				155068.9				138595.8			

Abbreviations: HR, heart rate; SBP, systolic blood pressure; DBP, diastolic blood pressure; β, coefficient estimates; SE, standard error; -2LL, 2LogLikelihood.

Significant difference at P < 0.05
